# The diagnostic performance of perfusion MRI for differentiating glioma recurrence from pseudoprogression: A meta-analysis

**DOI:** 10.1097/MD.0000000000006333

**Published:** 2017-03-24

**Authors:** Bing Wan, Siqi Wang, Mengqi Tu, Bo Wu, Ping Han, Haibo Xu

**Affiliations:** a Department of Radiology, Union Hospital of Tongji Medical College, Huazhong University of Science and Technology; b Department of Radiology, Zhongnan Hospital of Wuhan University, Wuhan, China.

**Keywords:** CBV, glioma recurrence, meta-analysis, perfusion MRI, pseudoprogression

## Abstract

**Background:**

The purpose of this meta-analysis was to evaluate the diagnostic accuracy of perfusion magnetic resonance imaging (MRI) as a method for differentiating glioma recurrence from pseudoprogression.

**Methods:**

The PubMed, Embase, Cochrane Library, and Chinese Biomedical databases were searched comprehensively for relevant studies up to August 3, 2016 according to specific inclusion and exclusion criteria. The quality of the included studies was assessed according to the quality assessment of diagnostic accuracy studies (QUADAS-2). After performing heterogeneity and threshold effect tests, pooled sensitivity, specificity, positive likelihood ratio, negative likelihood ratio, and diagnostic odds ratio were calculated. Publication bias was evaluated visually by a funnel plot and quantitatively using Deek funnel plot asymmetry test. The area under the summary receiver operating characteristic curve was calculated to demonstrate the diagnostic performance of perfusion MRI.

**Results:**

Eleven studies covering 416 patients and 418 lesions were included in this meta-analysis. The pooled sensitivity, specificity, positive likelihood ratio, negative likelihood ratio, and diagnostic odds ratio were 0.88 (95% confidence interval [CI] 0.84–0.92), 0.77 (95% CI 0.69–0.84), 3.93 (95% CI 2.83–5.46), 0.16 (95% CI 0.11–0.22), and 27.17 (95% CI 14.96–49.35), respectively. The area under the summary receiver operating characteristic curve was 0.8899. There was no notable publication bias. Sensitivity analysis showed that the meta-analysis results were stable and credible.

**Conclusion:**

While perfusion MRI is not the ideal diagnostic method for differentiating glioma recurrence from pseudoprogression, it could improve diagnostic accuracy. Therefore, further research on combining perfusion MRI with other imaging modalities is warranted.

## 1 Introduction

High-grade gliomas, which are classified by the World Health Organization based on invasive potential and increased proliferative capability, are the most common and lethal primary brain tumors in adults.^[1]^ Despite medical and scientific efforts over the past decades, current treatments remain dependent on neurosurgery, radiotherapy, and chemotherapy, which show a limited overall effect. Several studies have demonstrated that the median survival time of patients is only 3 to 9 months after the first tumor recurrence.^[2,3]^ Therefore, early identification of glioma recurrence may improve outcomes.

Generally, glioma recurrence is detected using imaging technology, such as computed tomography (CT) and magnetic resonance imaging (MRI).^[4]^ Radiation necrosis and other normal responses associated with surgical treatment may lead to mimicking of tumor recurrence, also known as pseudoprogression. Pseudoprogression and recurrence lesions have similar features on contrast-enhanced MRI or CT.^[4,5]^ As the 2 lesions require different treatment strategies and have totally different prognoses, accurate differentiation between recurrences and pseudoprogression lesions is critical. Recent developments in imaging technology have made it possible to monitor tumors at the metabolite and microvascular levels. Single-photon emission computed tomography (SPECT),^[6]^ positron emission tomography (PET),^[7,8]^perfusion CT,^[9]^ diffusion MRI,^[10,11]^ perfusion MRI, and magnetic resonance spectroscopy (MRS)^[12,13]^ are the imaging modalities in the clinical setting.

Perfusion MRI is an effective and advanced imaging method, which is widely used for qualitative diagnosis and postoperative follow-up of brain tumors, depending on information such as tumor blood volume and vascular permeability.^[14,15]^ Cerebral blood volume (CBV) reflects tumor angiogenesis and is the most important parameter of perfusion MRI. Relative CBV (rCBV), the ratio of blood volume in the lesion to that in the contralateral normal brain tissue, is often used instead of the absolute value. Some researchers have investigated the diagnostic value of rCBV in differentiating glioma recurrence from pseudoprogression. However, separately reported sensitivity, specificity, and cut-off values are significantly inconsistent. In this meta-analysis, we aimed to analyze the usefulness of rCBV for differentiating glioma recurrence from pseudoprogression.

## 2 Methods

### 2.1 Ethics statement

As this meta-analysis was performed based on the published data, ethical approval was not required.

### 2.2 Literature search

A comprehensive and systematic literature search was performed to identify relevant articles published before August 3, 2016 in the PubMed, Embase, Cochrane Library, and Chinese Biomedical databases. The keywords for the search were (“brain neoplasm” or “glioma” or “glioblastoma” or “astrocytoma” or “oligodendrocytoma” or “brain tumor” or “brain tumour” or “neuroectodermal tumor” or “neuroectodermal tumour” or “ependymoma” or “oligodendroglioma” or “neuroglioma” or “glial tumor” or “glial tumour”) and (“perfusion” or “PWI” or “CBV” or “cerebral blood volume” or “rCBV”) and (“MRI” or “MR” or “magnetic resonance”) and (“recurrence” or “tumor progression” or “postradiation” or “radiation necrosis” or “recurrent” or “radiation injury” or “pseudoprogression”). Articles in English or Chinese were chosen. Meanwhile, we also widely scanned references cited in the retrieved articles to find other potentially eligible articles.

### 2.3 Inclusion and exclusion criteria

The inclusion criteria were as follows: retrospective and prospective studies; studies based on clinical research in humans and regarding the assessment of rCBV for differentiating glioma recurrence from pseudoprogression; final diagnosis based on pathological or follow-up data; sufficient raw data was available to calculate true-positive, false-positive, false-negative, and true-negative values; more than 20 patients in total; in case of overlapping data, the study with the most cases. Review articles, letters, abstracts, comments, proceedings, and case reports were excluded. Articles where arterial spin labeling (ASL) was used as a perfusion MRI technique were also excluded.

Two authors assessed and identified potential articles based on the inclusion and exclusion criteria independently, and disagreement was resolved by arbitration by the third author.

### 2.4 Data extraction and quality assessment

Based on the inclusion criteria described above, the following types of characteristics were extracted from the articles: study characteristics (the name of first author, year of publication, source of publication, study design), patient characteristics (age, sex, numbers of population, and lesions), tumor status and treatment (glioma grade, radiation therapy type, and dose), and MRI technology (magnetic field strength, parameter, diagnostic threshold). The true-positive, false-positive, false-negative, and true-negative values were also noted.

The quality of included studies was assessed based on the quality assessment of diagnostic accuracy studies (QUADAS-2).^[16]^ The final list of included studies was decided by mutual agreement between all authors.

### 2.5 Statistical analysis

Individual studies have different thresholds leading to a variation in the sensitivities and specificities of diagnosis. The threshold effect can be identified visually by a “shoulder-arm” shape in the receiver operating characteristic curve (ROC) plane, or a strong positive correlation (*P* < 0.05) between the logit of sensitivity and the logit of (1-specificity).^[17]^ Cochran Q-statistic^[17]^ and inconsistency index (*I*-squared, *I*^2^)^[17]^ were measured to determine the heterogeneity of the studies. A *P* value less than 0.1 and an *I*^2^ value more than 50% indicated heterogeneity. In case of heterogeneity, a random-effects model was used, otherwise a fixed-effects model was used.^[18]^ Publication bias was evaluated visually based on symmetry of the funnel plot, while quantitative assessment involved the Deek funnel plot asymmetry test,^[19]^ and *P* < 0.1 indicated significant asymmetry.

Pooled sensitivity, specificity, positive likelihood ratio (PLR), negative likelihood ratio (NLR), diagnostic odds ratio (DOR), and their 95% confidence intervals (CI) were calculated as a whole, and were displayed as forest plots.^[17,20,21]^ The summary receiver operating characteristic curve (SROC), area under the curve (AUC), and Q∗ index (point at which the sensitivity and specificity are equal) were also calculated. AUC values of more than 80% represented the greatest potential for actual clinical application.

All statistical analyses were performed using MetaDisc (version 1.4), Review manager (version 5.3), and Stata (version 12.0) software.

## 3 Results

### 3.1 Study selection

**Figure 1:**
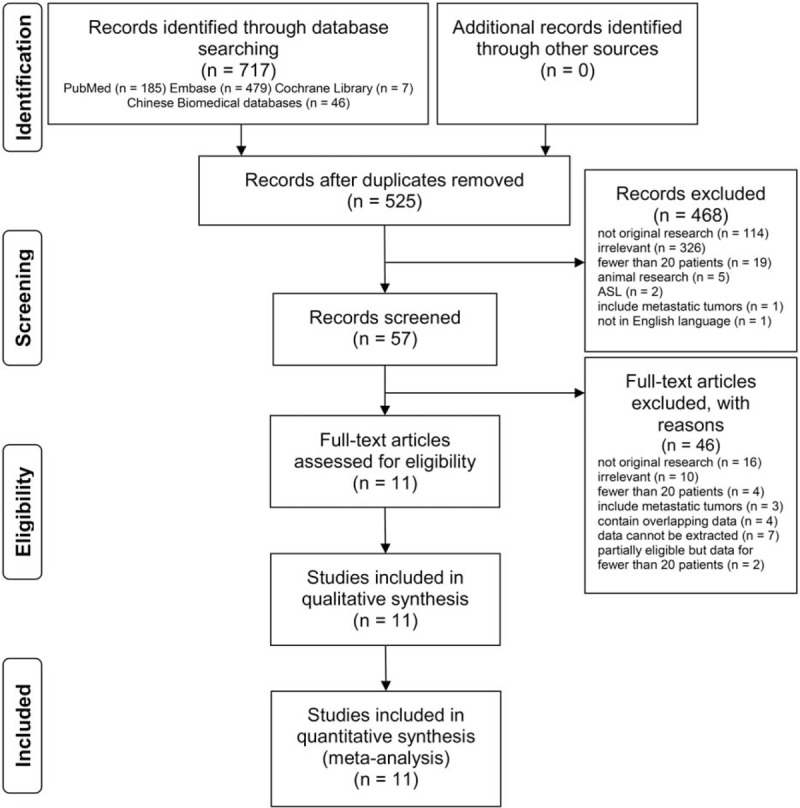
Flowchart of the study selection procedure. Ten retrospective and 1 prospective studies were included in the meta-analysis.

The comprehensive literature search yielded 717 articles, which included 185 articles from PubMed, 479 articles from Embase, 7 articles from the Cochrane Library, and 46 articles from the Chinese Biomedical database. After removing duplicate articles and reading the titles and abstracts, we identified 57 articles meeting the inclusion and exclusion criteria. The resulting full-text articles were assessed and 46 articles were excluded for the following reasons: not original research (n = 16), irrelevant (n = 10), fewer than 20 patients (n = 4), include metastatic tumors (n = 3), contain overlapping data (n = 4), data cannot be extracted (n = 7), and partially eligible but data for fewer than 20 patients (n = 2). Finally, 11 articles were included in this meta-analysis. A flowchart of the study selection procedure is shown in Figure 1.

### 3.2 Summary of included studies and quality assessment

**Figure 2:**
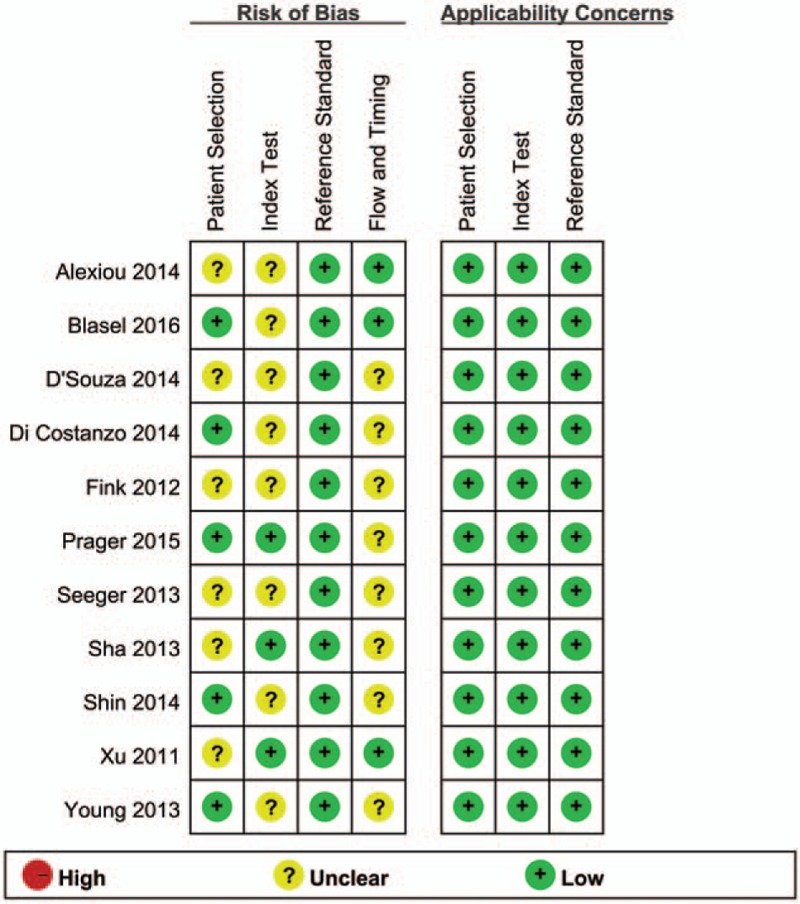
The methodological quality summary of the included studies using the quality assessment of diagnostic accuracy studies (QUADAS-2) tool. Green, red, and yellow circles indicate good, low, and unclear risk of bias, respectively.

**Figure 3:**
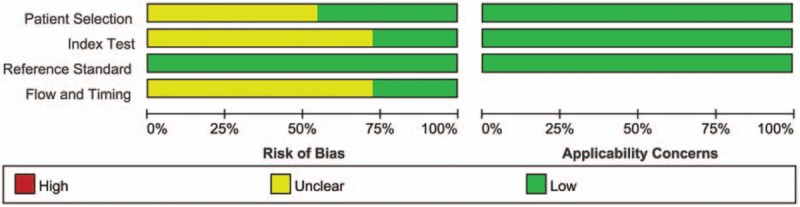
The methodological quality graph of the included studies using quality assessment of diagnostic accuracy studies (QUADAS-2) tool. Green, red, and yellow bars proportionly indicate good, low, and unclear risk of bias, respectively.

Patients’ characteristics, and the methodological quality as well as technical imaging and parameter values of the extracted studies are shown in Table 1.^[10,11,22–30]^ A total of 416 patients and 418 lesions were included in the 11 articles, which were published between 2011 and 2016. Patients’ ages ranged from 9 to 84 years. Ten studies were retrospective, while 1 was prospective. According to the World Health Organization glioma classification, most tumors were classified as high-grade gliomas. All patients had histologic or clinical follow-up findings that could be used as a reference standard to differentiate glioma recurrence from pseudoprogression. The MRI field strength was 1.5 T and 3.0 T. Other imaging modalities such as diffusion-weighted imaging, MRS, diffusion tensor imaging, technetium-99m SPECT, and methyl-11C-l-methionine (11c-MET) PET/CT were also used in some articles. The summary of the quality assessment of included studies with regard to risk of bias and applicability concerns is presented in Figure 2. Figure 3 displays a graph showing the overall proportion of studies with low, high, or unclear risk of bias. In summary, the quality of included studies met requirements for this meta-analysis.

**Table 1:**
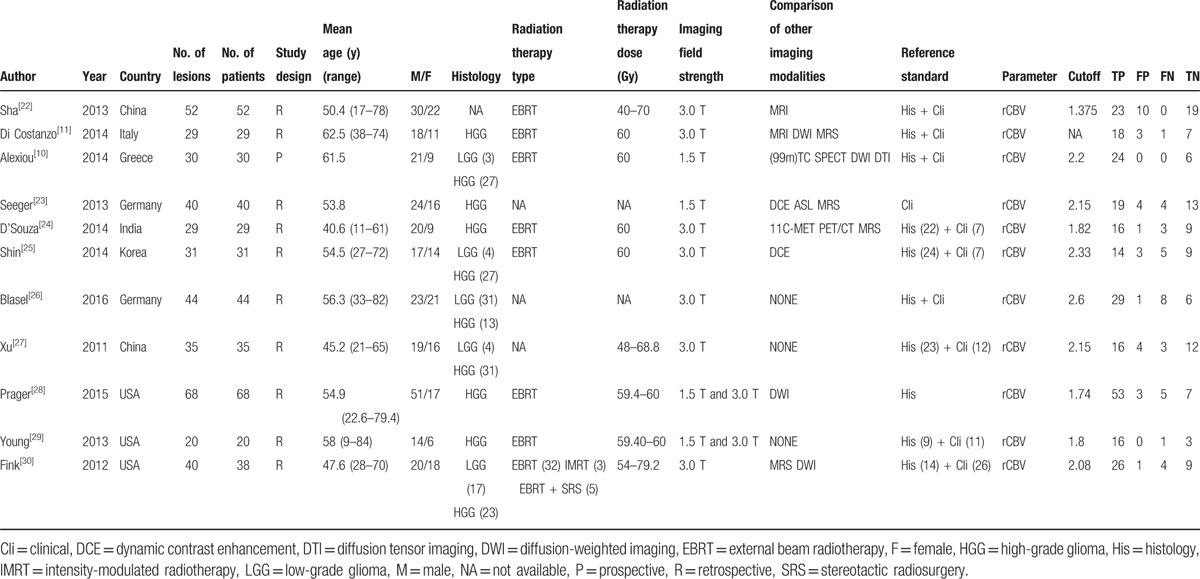
Main characteristics of the 11 studies included in the meta-analysis.

### 3.3 Quantitative synthesis

**Figure 4:**
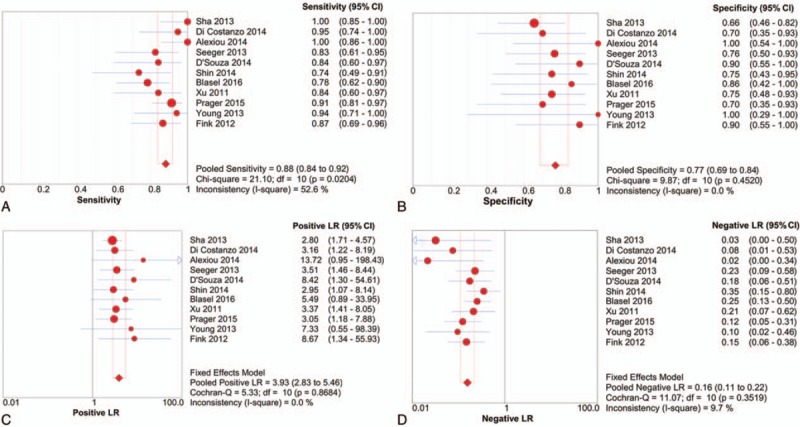
Forest plots of sensitivity (A), specificity (B), positive likelihood ratio (C), and negative likelihood ratio (D) of perfusion-weighted imaging for differentiating glioma recurrence from pseudoprogression. The pooled sensitivity, specificity, positive likelihood ratio, and negative likelihood ratio were 0.88, 0.77, 3.93, and 0.16, respectively.

**Figure 5:**
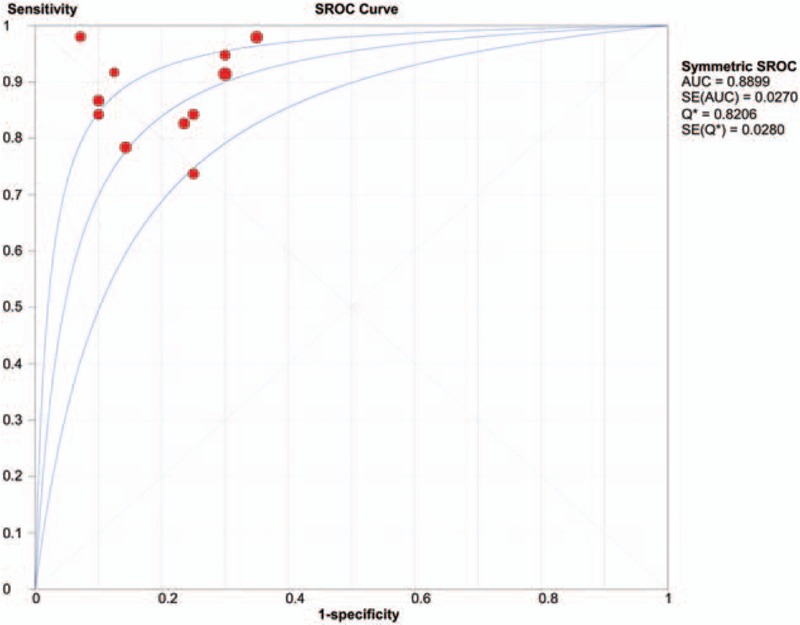
Summary receiver-operation characteristic curve for perfusion-weighted imaging differentiating glioma recurrence from pseudoprogression. The area under the curve was 0.8899, suggesting good but not excellent diagnostic accuracy.

A threshold effect was absent as evidenced by the absence of a “shoulder-arm” shape in the ROC plane. Further analysis showed that the Spearman Correlation Coefficient between the logit of sensitivity and the logit of (1-specificity) was 0.041 (*P* = 0.904), confirming that there was no obvious threshold effect among individual studies. There was heterogeneity in the sensitivities of the 11 included studies (*I*^2^ = 52.6%, *P* = 0.02), so a random-effects model was used. The pooled sensitivity was 0.88 (95% CI 0.84–0.92). The fixed-effects model was used to pool specificity, PLR, NLR, and DOR when data were not heterogeneous. The pooled values were 0.77 (95% CI 0.69–0.84), 3.93 (95% CI 2.83–5.46), 0.16 (95% CI 0.11–0.22), and 27.17 (95% CI 14.96–49.35), respectively. The forest plots of sensitivity, specificity, PLR, and NLR are shown in Figure 4. The AUC under the SROC was 0.8899 (Figure 5).

### 3.4 Sensitivity analysis and publication bias

Among the 11 included studies, 7 studies used 3.0 T field strength MRI, 2 studies used 1.5 T field strength MRI, and the remaining 2 studies used both 1.5 T and 3.0 T for perfusion MRI. There was no notable threshold effect in 7 studies utilizing 3.0 T field strength (*P* = 0.323), based on sensitivity analysis. The pooled weighted sensitivity, specificity, PLR, NLR, and DOR, and relevant 95% CI of 3.0 T field strength studies were as follows: 0.86 (95% CI 0.79–0.91), 0.76 (95% CI 0.66–0.84), 3.77 (95% CI 2.58–5.52), 0.17 (95% CI 0.11–0.26), and 26.3 (95% CI 12.35–56.00), respectively. The AUC under the SROC was 0.8907. The overall statistical parameters did not change when only the 3.0 T field strength studies were included. Therefore, we infer that the meta-analysis results are relatively stable and credible.

**Figure 6:**
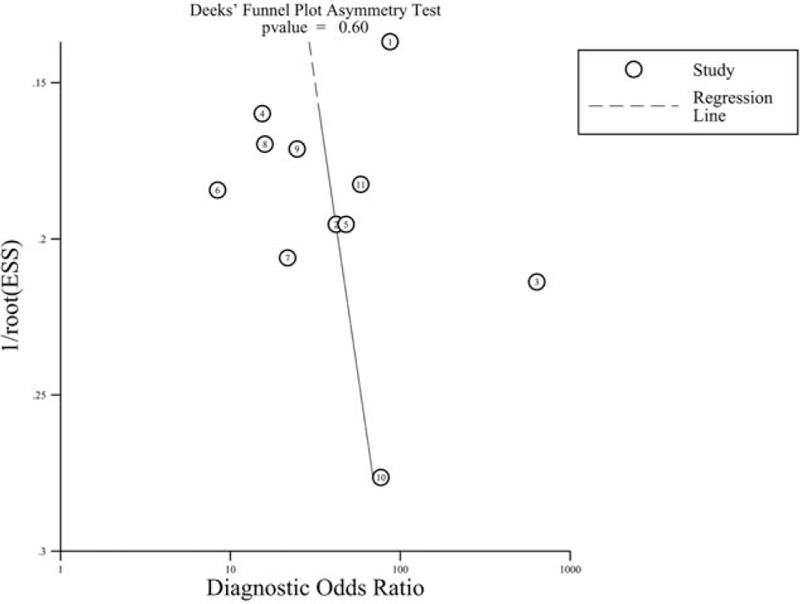
Publication bias tested using Deek funnel plot asymmetry test. *P* = 0.6 indicated no obvious publication bias.

The funnel plots of the 11 studies, which were scatter plots of DOR against 1/(effective sample size)^1/2^, were symmetrical. Moreover, the Deek test showed no obvious publication bias (*P* = 0.6; Figure 6).

## 4 Discussion

The prevalence of pseudoprogression in glioma patients is about 20% to 30% according to previous studies.^[31,32]^ An erroneous diagnosis of glioma recurrence or pseudoprogression may lead to interruption of the standardized first-line therapy and/or unnecessary surgery. Unfortunately, traditional surgery, radiation, and chemotherapy can lead to contrast-enhanced lesions and surrounding edema as in glioma progression.^[32]^ Although many attempts have been made, it is still a challenge to accurately diagnose newly enhanced lesions using conventional contrast-enhanced MRI.^[33,34]^ Perfusion MRI is an important advanced imaging technology that can provide information about tumor neoangiogenesis and microvascular leakiness.^[35]^ Dynamic susceptibility contrast MRI, which is based on tracking the passage of a contrast agent by dynamic imaging, and ASL, which does not rely on a gadolinium-based tracer, are 2 different perfusion MRI methods. Due to the drawback of an inherently low signal-to-noise ratio, difficulties with image postprocessing, and lack of guidelines for interpretation of ASL,^[36,37]^ we selected dynamic susceptibility contrast MRI, which is used more commonly in clinical practice, as the only perfusion method in this meta-analysis. Some studies have investigated the significance of a higher value of the perfusion MRI parameter, rCBV, in tumor recurrence to obtain better diagnostic accuracy.^[26,32]^ Newly formed immature blood vessels of recurrent tumors can result in increased blood volume and vascular permeability, resulting in a significantly higher rCBV value. We performed a meta-analysis to determine the value of using perfusion MRI for differentiating between glioma recurrence and pseudoprogression.

We first assessed the threshold effect of 11 individual studies through a ROC space. The lack of a “shoulder-arm” shape and the Spearman Correlation Coefficient value (0.041) indicated that different cut-off points had a mild effect on accuracy of individual studies. We also tested the heterogeneity using Cochran Q statistic and *I*^2^. The results showed obvious heterogeneity in the pooled sensitivity, and homogeneity in the pooled specificity, PLR, NLR, and DOR. The rCBV diagnostic accuracy value of the DOR was 27.17, suggesting that this be a useful method to identify glioma recurrence. A PLR of 3.93 in this meta-analysis revealed that patients with a rCBV higher than the cutoff are 3 times more likely to have a glioma recurrence. In contrast, an NLR of 0.16 suggested that an rCBV lower than the cutoff value had an 84% chance of being glioma pseudoprogression. Notably, this meta-analysis indicated that the rCBV value was not accurate enough to distinguish glioma recurrence from pseudoprogression. The AUC of the SROC was 0.8899, suggesting good but not excellent diagnostic accuracy. Results of the sensitivity analysis using magnetic field strength as a variable suggested that the meta-analysis results are reliable. Although only English and Chinese articles are included, the funnel plot and Deek test indicated that there was no significant publication bias in this meta-analysis.

Several studies have examined the value of perfusion MRI for distinguishing glioma recurrence from pseudoprogression, but the results are inconsistent. D'Souza et al^[24]^ reported that the sensitivity, specificity, and accuracy of 11c-MET PET/CT in identifying tumor recurrence were 94.7%, 80%, and 89.6%, respectively, whereas those of advanced MRI techniques were 84.2%, 90%, and 86.2%, respectively. Thus, 11c-MET PET/CT seemed to be more sensitive compared with the more specific advanced MRI. Matsusue et al^[12]^ found that optimum thresholds of the apparent diffusion coefficient (ADC, 1.30) and rCBV (2.10) combined with the choline-to-creatine ratio (Cho/Cr, 1.29) or choline-to-N-acetylaspartate ratio (Cho/NAA, 1.06) produced diagnostic accuracies less than 90%. Di Costanzo et al^[11]^ reported that the diagnostic accuracy of the metabolite ratios, Cho/Cr, to discriminate between glioma recurrence and pseudoprogression was 79.3%. Additionally, the combinations of Cho/Cr and ADC; Cho/Cr and rCBV; Cho/Cr, ADC and rCBV improved the diagnostic accuracy to 86.2%, 89.7%, and 96.6%, respectively. Seeger et al^[23]^ showed among the parameters in MRS, Cho/Cr offers the best diagnostic accuracy (sensitivity 70%, specificity 78.6%), and diagnostic accuracy could be increased to 82.5% by also considering rCBV. In this meta-analysis, the PLR and NLR values are not sufficient for clinical utility despite rCBV having a good diagnostic accuracy. Hence, combining rCBV with other imaging methods could be better for differentiating glioma recurrence from pseudoprogression. However, multimodal imaging is more expensive, time consuming, and may need extra contrast agent. It is also hard to decide the relative value when the outcome of each technique is inconsistent. Furthermore, there is no standard examination procedure to get a more reliable result. As techniques develop and more research is conducted, multimodal imaging may become more widely used. Chuang et al^[38]^ previously performed a meta-analysis to differentiate radiation-induced necrosis from recurrent brain tumors by magnetic resonance perfusion and spectroscopy. They included patients with primary brain tumor as well as those with brain metastases. Their findings showed added clinical usefulness irrespective of the type of brain tumor. However, identification of glioma recurrence was not accurate because different types of primary tumors or brain metastases have distinct biological entities. Shan et al^[39]^ also examined the value of MRS and perfusion MRI for diagnosing glioma recurrence by a meta-analysis and integrated all the perfusion parameters. The pooled sensitivity (0.84), specificity (0.84), PLR (5.51), NLR (0.19), DOR (28.09), and AUC (0.90) for perfusion MRI were different from this meta-analysis when only the CBV was included. All the above-mentioned weaknesses limit the application of multimodal imaging in daily clinical practice. As a simple and useful method for differentiating glioma recurrence from pseudoprogression, single imaging parameter is more preferable to guide the glioma treatment than multiple parameters.

This meta-analysis has some limitations. First, during treatment, the appearance of telangiectasis, an aneurysm, or vascular elongation may also cause an increase in rCBV. Meanwhile, microbleeding during radiation treatment can result in a decrease in rCBV in tumor recurrence.^[28,40]^ Thus, treatment-related changes can interfere with the real rCBV baseline. Second, methodological differences limit consistency in the included studies. The rCBV can be influenced by nonstandardized procedures. Although we excluded the influence of ASL technical factors, different MRI equipment, brands, field strength, scan parameters, therapy methods, frequency, and time intervals after treatment may lead to different results.^[33]^ Third, for various grades of gliomas, both histopathology and follow-up MRI were the standard diagnostic methods. Fourth, only English and Chinese language literatures were included in this meta-analysis, which might have resulted in missing some articles and induced potential publication bias. Finally, the included studies were mostly retrospective in design except for 1 that was prospective. Despite the inherent shortcomings described above, our analysis gives a quantitative analysis of rCBV for differentiating glioma recurrence from pseudoprogression.

In conclusion, rCBV significantly improves the diagnostic performance of glioma recurrence compared with conventional T1- and T2-weighted imaging, and contrast-enhanced MRI. The threshold value, rCBV, had moderately high diagnostic accuracy for differentiating glioma recurrence from pseudoprogression. Due to the limitations addressed above, additional studies with large sample sizes and standardized methodology would be required to achieve a more robust and credible result. Perfusion MRI combined with other imaging modalities such as diffusion MRI, MRS, SPECT, and PET should be researched further for introduction into routine clinical practice.

## Acknowledgments

The authors thank Editage (www.editage.com) for English language editing.
